# Longitudinal Pharmacokinetic-Pharmacodynamic Biomarkers Correlate With Treatment Outcome in Drug-Sensitive Pulmonary Tuberculosis: A Population Pharmacokinetic-Pharmacodynamic Analysis

**DOI:** 10.1093/ofid/ofaa218

**Published:** 2020-06-06

**Authors:** Frank Kloprogge, Henry C Mwandumba, Gertrude Banda, Mercy Kamdolozi, Doris Shani, Elizabeth L Corbett, Nadia Kontogianni, Steve Ward, Saye H Khoo, Geraint R Davies, Derek J Sloan

**Affiliations:** 1 Institute for Global Health, University College London, London, United Kingdom; 2 Malawi Liverpool Wellcome Trust Clinical Research Programme, Blantyre, Malawi; 3 Liverpool School of Tropical Medicine, Liverpool, United Kingdom; 4 Department of Microbiology, College of Medicine, University of Malawi, Blantyre, Malawi; 5 London School of Hygiene and Tropical Medicine, London, United Kingdom; 6 Department of Pharmacology, University of Liverpool, Liverpool, United Kingdom; 7 Institute of Global Health, University of Liverpool, Liverpool, United Kingdom; 8 School of Medicine, University of St Andrews, St Andrews, United Kingdom

**Keywords:** outcome, pharmacokinetics, pharmacodynamics, standard treatment, tuberculosis

## Abstract

**Background:**

This study aims to explore relationships between baseline demographic covariates, plasma antibiotic exposure, sputum bacillary load, and clinical outcome data to help improve future tuberculosis (TB) treatment response predictions.

**Methods:**

Data were available from a longitudinal cohort study in Malawian drug-sensitive TB patients on standard therapy, including steady-state plasma antibiotic exposure (154 patients), sputum bacillary load (102 patients), final outcome (95 patients), and clinical details. Population pharmacokinetic and pharmacokinetic-pharmacodynamic models were developed in the software package NONMEM. Outcome data were analyzed using univariate logistic regression and Cox proportional hazard models in R, a free software for statistical computing.

**Results:**

Higher isoniazid exposure correlated with increased bacillary killing in sputum (*P < *.01). Bacillary killing in sputum remained fast, with later progression to biphasic decline, in patients with higher rifampicin area under the curve (AUC)_0-24_ (*P < .*01). Serial sputum colony counting negativity at month 2 (*P < .*05), isoniazid *C*_MAX_ (*P < *.05), isoniazid *C*_MAX_/minimum inhibitory concentration ([MIC] *P < .*01), and isoniazid AUC_0-24_/MIC (*P < .*01) correlated with treatment success but not with remaining free of TB. Slower bacillary killing (*P < .*05) and earlier progression to biphasic bacillary decline (*P < .*01) both correlate with treatment failure. Posttreatment recurrence only correlated with slower bacillary killing (*P < .*05).

**Conclusions:**

Patterns of early bacillary clearance matter. Static measurements such as month 2 sputum conversion and pharmacokinetic parameters such as *C*_MAX_/MIC and AUC_0-24_/MIC were predictive of treatment failure, but modeling of quantitative longitudinal data was required to assess the risk of recurrence. Pooled individual patient data analyses from larger datasets are needed to confirm these findings.

## Teaser

Systemic exposure to isoniazid and rifampicin correlates with bacillary clearance from sputum and bacillary clearance from sputum correlates with tuberculosis treatment outcome. These findings show the potential of longitudinal PKPD biomarkers for trial design and clinical decision making.

Tuberculosis (TB) caused an estimated 1.2 million deaths worldwide from 10 million clinical cases in 2018 [1]. There is an urgent need for short, effective treatment. The current standard treatment of drug-sensitive TB remains long at 6 months, and efforts to shorten standard TB treatment have been unsuccessful [2–4]. Potent early antibacterial activity from short-course moxifloxacin-containing regimens did not translate into noninferior treatment success rates compared with standard of care regimens in Phase III clinical trials [2]. Ongoing efforts to tackle the problem include new trials of novel drug combinations (eg, SimpliciTB; ClinicalTrials.gov Identifier NCT03338621) and consideration of treatment individualization or stratification, targeting shorter treatment to the patients who are most likely to be cured (eg, TRUNCATE-TB; ClinicalTrials.gov Identifier NCT03474198). Both approaches would benefit from a biomarker that predicts final treatment outcome during the early weeks of treatment. This currently does not exist and treatment stratification is currently focused on baseline measurements. Progress would be accelerated by improved understanding of relationships between antibiotic exposure for individual antimicrobial drugs, serial quantitative bacterial load measurements, and clinical outcomes. Statistical and mathematical modeling has already enhanced our understanding of the aforementioned relationships [5–7]. Several pharmacodynamic (PD) and pharmacokinetic-pharmacodynamic (PKPD) models have been developed to study the correlation between drug concentrations and the bactericidal effect of treatment [6–11]. These models enable prediction of early drug effect, but analyses become difficult when bacterial loads fall below the limit of quantification. Month 2 and month 3 culture sputum culture conversion have been proposed as moderately good predictors of long-term treatment failure or relapse risk between treatment arms of early phase clinical trials [5]. However, the predictive value of culture conversion is weak for individual patient care [12], hampering robust translation to drug development and clinical programs.

Our overarching hypothesis is that integrating models describing correlations between individual antibiotic exposure and early antibacterial effect with models relating early antibacterial effect to treatment outcome will improve our understanding of treatment response and contribute to improved outcome predictions. The aim of this study was to explore predictors for treatment outcome using baseline demographic, steady-state plasma antibiotic exposure and serial sputum bacillary load data from drug-sensitive TB patients. Specific goals were to (1) fit pharmacokinetic (PK) models to concentration-time data for each of the first-line anti-TB drugs, (2) fit a population PKPD model to sputum bacterial load-time data, and (3) explore associations between early bacillary clearance from sputum and treatment success and recurrence of TB.

## MATERIALS AND METHODS

### Study Population and Study Design

Data from patients recruited to a longitudinal cohort study of clinical, pharmacological, and bacteriological responses to TB therapy conducted in Queen Elizabeth Central Hospital in Blantyre, Malawi were analyzed. The Liverpool School of Tropical Medicine and the College of Medicine Research Ethics Committee, University of Malawi gave ethical approval for this study. Longitudinal PD and outcome data from this patient cohort have been published [13]. The current study extends that work with PKPD analyses.

Consenting adults aged 16–65 years with sputum smear-positive pulmonary TB were eligible. Exclusion criteria included hemoglobin <6 g/dL, creatinine >177 μmol/L, total bilirubin >51 μmol/L, alanine transaminase >200 IU/L, clinical status suggestive of imminent mortality, pregnancy, prior TB treatment within 5 years, and concurrent corticosteroid therapy or baseline resistance to rifampicin and isoniazid using the Genotype MTBDRplus 2.0 line probe assay ([LPA] Hain Life Sciences). Patient characteristics have been reported previously [13]. Alcohol consumption (any beer or spirits) and smoking were reported as binary covariates based on practice at the time of study recruitment. Percentage of abnormal lung-field observed on baseline chest radiographs (CXR) was assessed by consensus of 2 independent readers. On binary variables (eg, presence/absence of cavities), the reviewers met to reach consensus on any discrepancies. On continuous variables (eg, percentage of lung-field affected), an average of the 2 scores was taken. Participants received daily fixed-dose combination tablets containing a standard first-line World Health Organization-approved regimen according to the *Malawi Ministry of Health National TB Control Programme Manual, 6th Edition, 2007*. The details are as follows: for the intensive phase of therapy, fixed-dose RZHE tablets (rifampicin 150 mg, isoniazid 75 mg, pyrazinamide 400 mg, ethambutol 275 mg) were used. For the continuation phase, RH tablets (rifampicin 150 mg, isoniazid 75 mg) were used. Weight bands for both phases were as follows: 2 tablets, 30–37 kg; 3 tablets, 37–54 kg; 4 tablets, 54–74 kg; and 5 tablets, >74 kg. Adherence was monitored by direct questioning and pill counts. All patients had point-of-care human immunodeficiency virus (HIV) serology. Antiretroviral therapy was provided per national protocols.

### Clinical Endpoint Definitions

An endpoint of stable cure was reported for patients who were clinically well with 2 consecutive negative sputum cultures by the end of treatment (EOT) and no TB recurrence in the subsequent 12 months. Treatment failure was reported for patients with positive sputum culture at EOT. Tuberculosis recurrence was reported for patients who appeared to be cured at EOT but re-presented with TB disease, ie, reinfection or relapse, in the next 12 months. The LPA was repeated on positive EOT or posttreatment *Mycobacterium tuberculosis* (*Mtb*) isolates from treatment failures or TB recurrences, respectively.

### Bacterial Load Measurement

Patients had overnight sputum samples collected on day 0, 2, 4, 7, 14, 28, 49, and 56 of treatment. Auramine-phenol microscopy was done on direct and concentrated sputum smears at all time points. Two 1-mL aliquots were used for sputum bacillary load measurement on solid (serial sputum colony counting [SSCC]) and liquid (Mycobacterial Growth Indicator Tube [MGIT]) culture. All patients submitted spot sputum samples after 5 months of therapy (EOT samples) to assess bacteriological cure. Those with ongoing or recurrent symptoms submitted posttreatment samples to test for relapse. Standard bacteriological methods for SSCC (results reported as log_10_ colony-forming units [CFU]/mL) and MGIT (results reported as days-to-positivity but analyzed in this paper as a binary positive/negative result at month 1 and 2) were as previously described [13]. Only patients who contributed at least 2 bacterial load measurements could be included in PKPD models, because a bacterial clearance rate cannot be calculated from a single measurement.

### Minimum Inhibitory Concentration Assays

*Mycobacterium tuberculosis* isolates from baseline sputum cultures of all patients were stored at −70^o^C for bioanalysis on UKMYC Sensititre plates (Thermo Fisher Scientific). Plates for this project were custom-configured by the manufacturer; the wells contained dry microdilutions of lyophilized antibiotics across concentrations, which allowed careful MIC titration within the drug-susceptible range (2 antibiotic-free control wells; doubling dilutions from 16 to 0.015 mg/L for rifampicin and isoniazid; doubling dilutions from 8 to 0.25 mg/L for ethambutol).

*Mycobacterium tuberculosis* isolates were revived in Middlebrook 7H9 broth, then subcultured onto Middlebrook 7H11 agar. Colonies were emulsified in 0.2% saline-tween with glass beads and vortexed for 30 seconds. Turbidity was adjusted to 0.5 McFarland Standard. One hundred microliters from each suspension were transferred into 11 mL Middlebrook 7H9 broth, supplemented with oleic acid-albumin-dextrose-catalase, to give an inoculum of 10^5^ CFU/mL. One hundred microliters of this material from each isolate were inoculated, in duplicate, into plate wells covering all concentrations of all drugs. Plates were covered with permanent plastic seals and incubated at 37°C in 5% CO_2_. Plates were checked for contamination at 24 and 48 hours, then monitored at 10, 14, and 21 days. Results were read when growth was clearly visible in the antibiotic-free control wells. For each antibiotic, the lowest concentration with no visible growth was considered to be the MIC. Each plate was read by 2 independent readers. The MIC result recorded by the first reader was the test result. The second reader’s result was used to assess interreader agreement.

### Antibiotic Plasma Concentration Measurement

Blood collections to measure steady-state isoniazid, rifampicin, pyrazinamide, and ethambutol concentrations were undertaken on day 14 or 21 of treatment. Patients attended the study clinic at 7:30 am, after an overnight fast. Samples were collected predose and then 2 and 6 hours after medication. To allow patients to return home before nightfall, 6 hours postdose was the latest sampling timepoint. Similar field-based clinical studies in southern Africa [14] have deployed similar collection strategies. Plasma was separated by centrifugation and stored at −70°C until batched analysis. Rifampicin, isoniazid, and ethambutol concentrations were determined using previously published liquid chromatographic/tandem mass spectrometry methods [15, 16] with appropriate internal standards validated to internationally recognized acceptance criteria as previously described [17]. Pyrazinamide concentrations were measured by high-performance liquid chromatography using an ultraviolet visible absorption detector.

### Pharmacokinetics

Population PK models were developed using the Stochastic Approximation Expectation Method in the Fortran-based software package NONMEM [18] with a g95 Fortran compiler. Empirical Bayes area under the curve (AUC)_0-24_ and *C*_MAX_ estimates were derived for each patient and evaluated as predictors on bacillary clearance and treatment outcome. Details on PK model building can be found in the Supplementary Materials.

### Pharmacokinetics-Pharmacodynamics

Bacillary clearance from sputum can be captured using monophasic (Figure 2, profile A) or biphasic models (Figure 2, profile B and C). Our PKPD model used the parameter LAM to represent the slope of a monophasic bacterial decay curve or the first phase of a biphasic bacterial decay curve (Figure 2). For biphasic clearance, the magnitude of decline in bacterial clearance was parameterized in this model by BETA, and the time it takes to switch from fast killing to slow killing was parameterized using the parameter t_1/2_ (Figure 2). Details on PKPD model building can be found in the Supplementary Materials.

*P* values of .05 and .01 were used as cutoffs in the forward inclusion and backward elimination step of stepwise covariate model building. Covariates, to be evaluated on PKPD model parameters, were selected based on physiological plausibility and a correlation matrix with post hoc pharmacodynamic parameter estimates from the baseline model. The following parameters were consequently evaluated on baseline sputum bacillary load using an exponential relationship: age, body mass index (BMI), percentage of abnormal lung-field on CXR, and baseline bilirubin and creatinine concentrations. Isoniazid and rifampicin AUC_0-24_ and *C*_MAX_, BMI, baseline serum bilirubin and creatinine concentrations, HIV infection, current alcohol consumption, and treatment adherence were evaluated on θ_LAM_. The AUC_0-24_ and *C*_MAX_ of all 4 study drugs, age, BMI, percentage of abnormal lung-field on CXR, baseline bilirubin, and baseline creatinine were evaluated on θ_t1/2_. The AUC_0-24_ and *C*_MAX_ of all 4 study drugs, age, BMI, percentage of abnormal lung-field on CXR, baseline bilirubin, and creatinine concentrations were evaluated on θ_BETA_. The MIC-adjusted isoniazid and rifampicin AUC_0-24_ were evaluated on a subset of patients with available MIC data. The AUC_0-24_/MIC was substituted for AUC_0-24_ equivalents in the final model, and statistical significance was tested using a backward elimination approach.

Empirical Bayes estimates for parameters LAM, BETA, and t_1/2_ were derived for each patient and evaluated as predictors for treatment outcome.

### Study Outcome

Univariate logistic regression was used to investigate the role of clinical, PK, and bacteriological factors as explanatory variables to predict stable cure and failure. A Cox-proportional hazard model was used to investigate the role of clinical, PK, and bacteriological factors as explanatory variables to predict recurrence among patients who had stable cure at EOT. Bacteriological factors were categorized as static measurements (eg, sputum smear and culture [SSCC or MGIT] conversion to negative at end of month 1 or 2) or dynamic PKPD-model-derived parameters (eg, LAM, t_1/2_, and BETA). Correlations between MIC-adjusted isoniazid, rifampicin, or ethambutol exposure (*C*_MAX_ or AUC_0-24_) and outcome (failure or recurrence) were evaluated on patients with available MIC data.

## RESULTS

Plasma antibiotic concentration-time data were available from 154 patients (Table 1). Fifty-two patients contributed fewer than 2 bacterial load samples leaving 102 for the PKPD analysis. Seven patients withdrew from the study before reaching EOT, 3 upon the patients’ request, 3 according protocol, and 1 patient was lost to follow-up, leaving 95 patients for the treatment failure outcome analysis. Five patients failed treatment. Among the 90 successfully treated patients, 1 withdrew from the study immediately after EOT and consequently did not participate in the follow-up phase, leaving 89 patients for recurrence outcome analysis. Among these patients, 78 remained TB free and 4 had recurrent infection. Seven patients were censored as 5 were lost to follow-up, and 2 died of non-TB causes during posttreatment follow-up (Supplementary S1 Figure).

**Table 1.** Demographic Summary of the Pharmacokinetic, Pharmacodynamic, and Outcome Data in Median (Range)

	Pharmacokinetic Data	Pharmacodynamic Data	Outcome Data; Cure vs. Failure/Recurrence
Sample size (*n*) [failure/recurrence]	154	102	95 [5]/89 [4]
Age (years)	30 [17–61]	30 [17–60]	30 [17–51]/31 [17–51]
Body weight (kg)	52 [34–74]	53 [35–74]	52 [35–74]/52 [35–74]
Height (m)	1.675 [1.5–1.84]	1.675 [1.5–1.82]	1.67 [1.5–1.82]/1.66 [1.5–1.82]
BMI (kg/m^2^)	18.505 [13.17–29.27]	18.94 [13.17–25]	18.37 [13.17–25]/18.36 [13.17–25]
CD4 (cells/mm^3^)	168 [6–783]	185.5 [6–616]	198 [6–539]/198 [8–539]
Haemoglobin (g/dl)	10.9 [5.9–18.7]	10.6 [5.9–18.7]	10.45 [5.9–18.7]/10.45 [5.9–18.7]
White blood cell count (cells/mm^3^)	6.5 [1.4–21.4]	6.9 [2.5–14]	6.9 [2.5–14]/7 [2.5–14]
Platelets (cells/mm^3^)	339 [44–922]	374 [109–922]	373 [109–922]/373 [109–922]
Urea (umol/l)	3.35 [1.9–21.9]	3.3 [1.9–9.2]	3.35 [1.9–9.2]/3.35 [1.9–9.2]
Creatinine (umol/l)	59.5 [29–117]	60 [29–111]	59 [29–111]/59 [29–111]
Bilirubin (umol/l)	8 [1–50]	7.5 [1–32]	7.65 [1–32]/7.65 [1–32]
Alatinine (IU/l)	20 [7–190]	19 [7–190]	19 [7–190]/19 [7–190]
Male (*n*) [%]	107 [69]	75 [74]	69 [73]/63 [71]
HIV (*n*) [%]	89 [58]	59 [58]	54 [57]/50 [56]
Alcohol (*n*) [%]	49 [32]	33 [32]	30 [32]/27 [30]
Smoking (*n*) [%]	17 [11]	14 [14]	13 [14]/11 [12]
No missed doses (*n*) [%]	139 [90]	94 [92]	89 [94]/84 [94]
Missed doses (*n*) [%]	13 [8]	7 [7]	6 [6]/5 [6]
Missing adherence information (*n*) [%]	2 [1]	1 [1]	0 [0]/0 [0]
MGIT status negative at Month 2 (*n*) [%]	88 [57]	56 [55]	53 [52]/50 [68]
MGIT status missing at month 2 (*n*) [%]	32 [21]	17 [17]	15 [15]/15 [20]
SSCC status negative at month 2 (*n*) [%]	108 [70]	74 [73]	71 [70]/69 [96]
SSCC status missing at month 2 (*n*) [%]	39 [25]	21 [21]	17 [17]/17 [24]
Smear status negative at month 2 (*n*) [%]	115 [75]	79 [77]	74 [73]/69 [83]
Smear status missing at month 2 (*n*) [%]	18 [12]	8 [8]	6 [6]/6 [7]
MIC (*n*) [%]	65 [42]	50 [49]	47 [49]/42 [47]
Isoniazid (mg/l)	0.015 [0.015–0.06]	0.015 [0.015–0.06]	0.015 [0.015–0.06]/0.015 [0.015–0.03]
Rifampicin (mg/l)	0.015 [0.015–0.06]	0.015 [0.015–0.06]	0.015 [0.015–0.06]/0.015 [0.015–0.06]
Ethambutol (mg/l)	0.5 [0.5–2]	0.5 [0.5–2]	0.5 [0.5–2]/0.5 [0.5–2]

MGIT, Mycobacteria growth indicator tube; SSCC, Serial Sputum Colony Counting; MIC, Minimum Inhibitory Concentration.

Ninety percent of patients reported no missed treatment doses, 13 patients reported missed doses and 2 patients had missing adherence information. All patients had rifampicin- and isoniazid-susceptible TB at baseline. The MICs for isoniazid, rifampicin, and ethambutol, available from 47 patients with clinical outcome data, confirmed that the *Mtb* isolates were extremely sensitive to the antibiotics used (Table 1). Of the 5 treatment failures, 1 had isoniazid and rifampicin resistance detected on LPA at EOT, suggesting acquisition of multidrug-resistant TB during therapy. Of the 4 TB recurrences, 1 had isoniazid monoresistance detected at symptomatic re-presentation, suggesting either acquisition of resistance on treatment or reinfection with an isoniazid monoresistant strain. A baseline profile of the study cohort is shown in (Table 1).

### Pharmacokinetics

**Figure 1.**
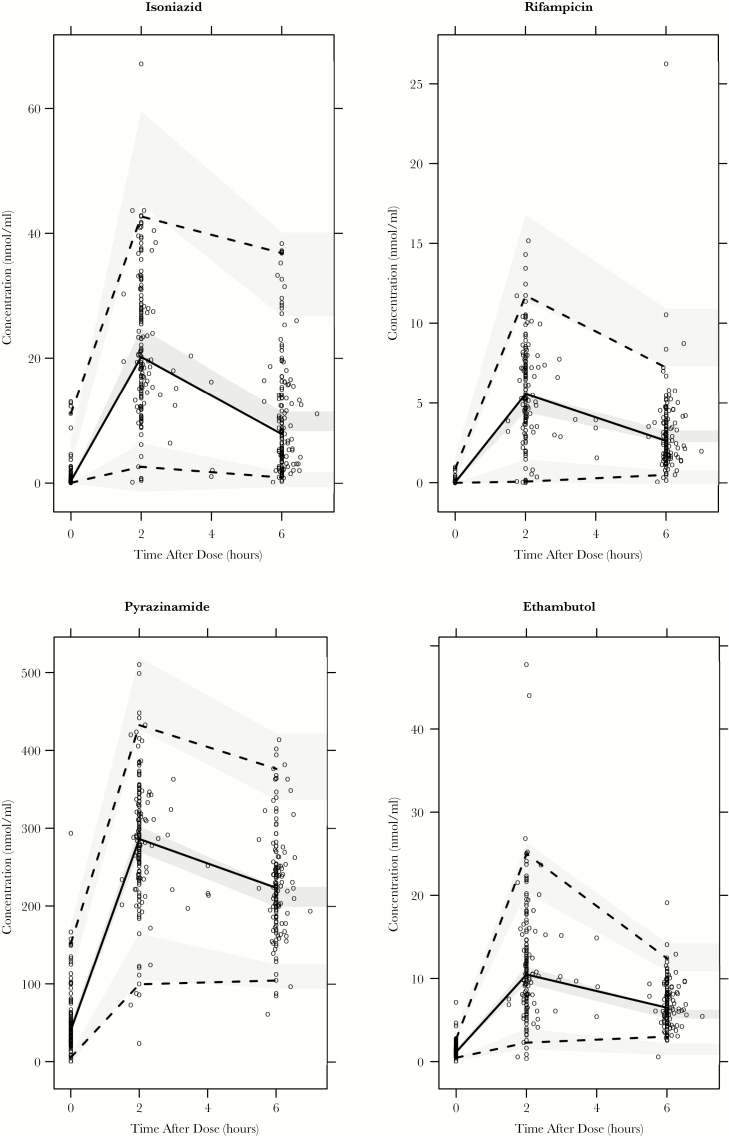
Simulation based (n = 2000) visual predictive checks for isoniazid (top left), rifampicin (top right), pyrazinamide (bottom left), and ethambutol (bottom right). Open circles represent observations, solid and dashed black lines represent observed 2.5, 50th, and 97.5 percentiles. Shaded areas represent the 90% confidence intervals around the simulated 2.5, 50th, and 97.5 percentiles.

Visual predictive checks and goodness-of-fit diagnostics for the isoniazid, rifampicin, pyrazinamide, and ethambutol models showed adequate predictive performance (Figure 1, Supplementary S2 and S3 Figures and S1 Table). Model-derived median *C*_MAX_ estimates and ranges for the study cohort were as follows: isoniazid, 3.24 (2.19–5.50) mg/L; rifampicin, 4.35 (2.30–12.30) mg/L; pyrazinamide, 40 (25–63) mg/L; and ethambutol, 2.30 (1.43–4.40) mg/L. Median AUC_0-24_ estimates and ranges were as follows: isoniazid, 18.83 (6.97–71.20) hxmg/L; rifampicin, 29.10 (14.50–119.00) hxmg/L; pyrazinamide, 419 (210–2014) hxmg/L; and ethambutol 18.50 (12.80–38.50) hxmg/L.

### Pharmacokinetics-Pharmacodynamics

**Figure 2.**
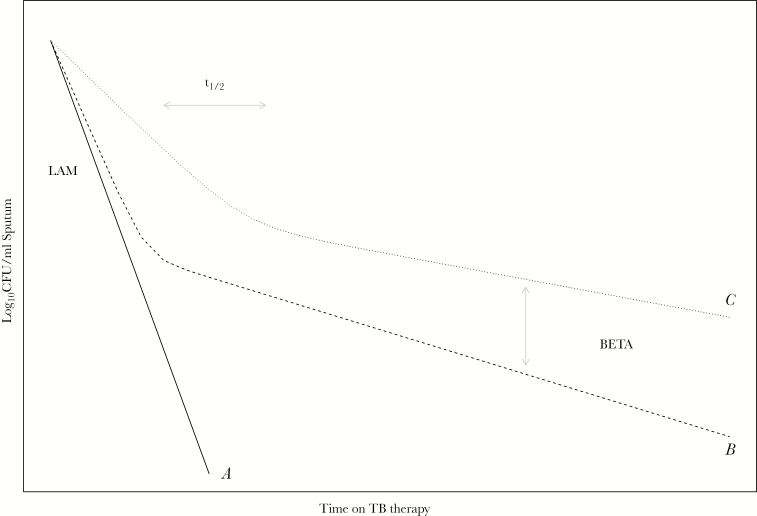
Visualization of pharmacokinetic-pharmacodynamic model characteristics. The solid line (A) illustrates monophasic bacillary clearance from sputum with a clearance rate represented by LAM. The dashed (B) and dotted (C) lines illustrate biphasic bacillary clearance trajectories; LAM represents the initial clearance rate (faster in B than C line), BETA represents the magnitude of decreased bacillary clearance (larger in C than B), and t_1/2_ represents the time it takes to switch from fast to slow killing (earlier in B than C). CFU, colony-forming units.

**Figure 3.**
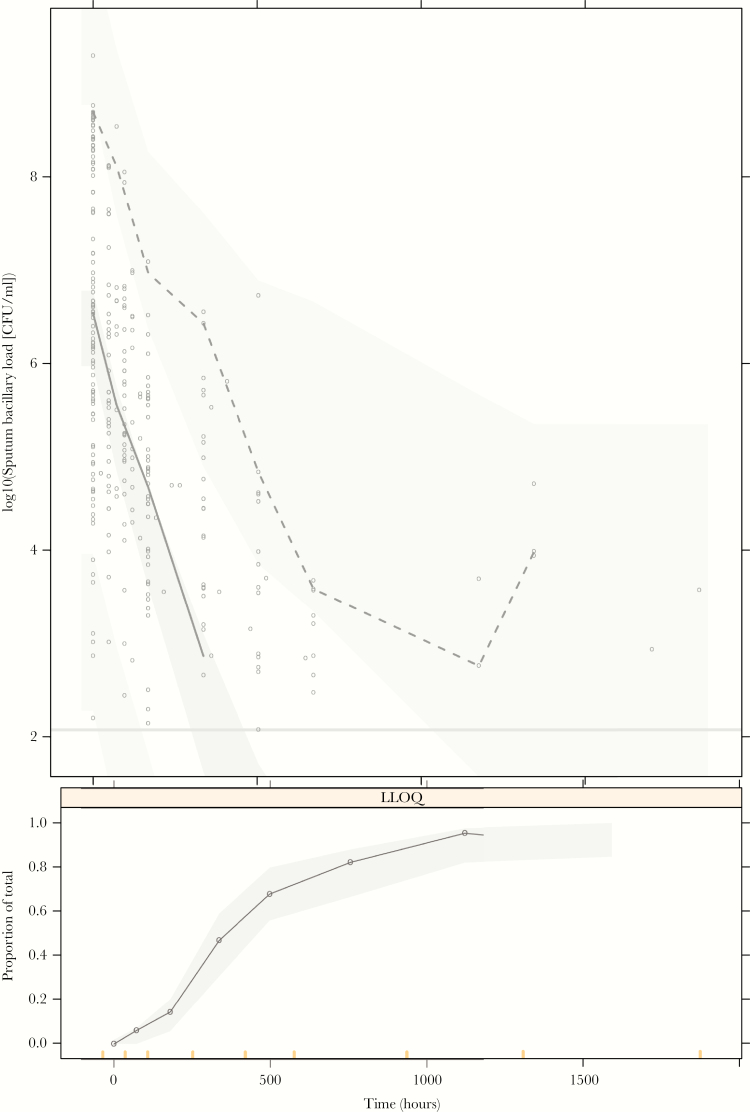
Simulation-based (n = 2000) visual predictive checks for the pharmacokinetic-pharmacodynamic model. Open circles in the top panel represent observations, and solid and dashed black lines represent observed 50th and 97.5 percentiles, respectively. Shaded areas in the top panel represent the 90% confidence intervals around the simulated 2.5, 50th, and 97.5 percentiles. The dots and solid line in the bottom panel represent the observed proportion of samples below the limit of quantification, and the shaded area represents the corresponding 90% confidence interval of proportion samples below limit of quantification produced by the model. Bars at the bottom of the lower panel indicate the binning windows. CFU, colony-forming units; LLOQ, lower limit of quantification.

**Figure 4.**
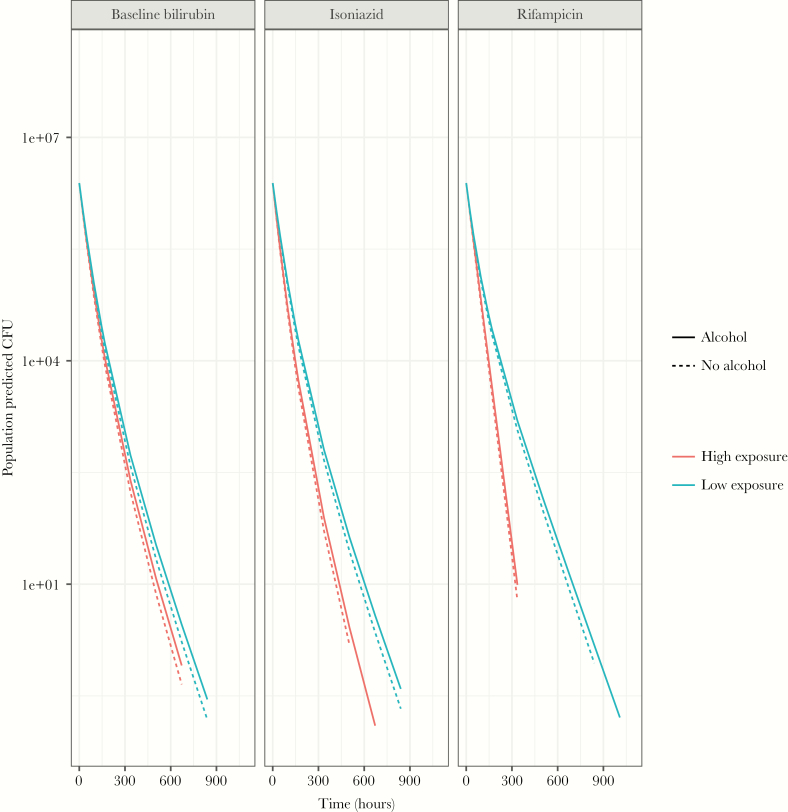
Visualization pharmacokinetic-pharmacodynamic covariate effects (baseline bilirubin-LAM; isoniazid-LAM; rifampicin-t_1/2_ and alcohol consumption-LAM]). High and low exposure refers to highest and lowest values in the study population: 1–32 μmol/L, 54.9–515 hrxμmol/L, and 19.9–145 hrxμmol/L for baseline bilirubin, isoniazid, and rifampicin, respectively. CFU, colony-forming units.

Isoniazid AUC_0-24_ at steady state and baseline serum bilirubin concentrations positively correlated (*P < .*01) in an exponential relation with LAM (Figure 2) in the PKPD model (Figure 3, Supplementary S4 and S5 Figures), resulting in a steeper bacillary kill-curve in sputum with higher isoniazid exposures and baseline bilirubin concentrations (Figure 4, Supplementary S2 Table). Rifampicin AUC_0-24_ at steady state in an exponential relation positively correlated (*P < .*01) with t_1/2_ (Figure 2), resulting in more pronounced biphasic kill-curves with lower rifampicin concentrations (Figure 4, Supplementary S2 Table). Alcohol consumption correlated with a slower LAM, resulting in a steeper bacillary kill-curve in sputum in nonalcohol consuming patients (Figure 4, Supplementary S2 Table).

Stepwise backward exclusion of isoniazid AUC_0-24_/MIC, when replacing AUC_0-24_, as covariate on LAM resulted in significant worsening of the model fit on a subset of the patients that had MICs reported (Supplementary S6 Figure and S3 Table). Stepwise backward exclusion of rifampicin AUC_0-24_/MIC, when replacing AUC_0-24_, as covariate on t_1/2_ resulted in a model that did not converge on the same subset of patients (Supplementary S6 Figure and S3 Table). As per protocol, MIC-adjusted PK parameters for ethambutol were not evaluated here because neither MIC unadjusted ethambutol AUC_0-24_ nor *C*_MAX_ was significantly correlated with LAM, t_1/2_, or BETA.

### Study Outcome

From the static bacteriological treatment response measurements, only SSCC negativity at month 2 correlated with the likelihood of treatment success at EOT (Tables 2). Among the PK variables, higher isoniazid *C*_MAX_, *C*_MAX_/MIC, and AUC_0-24_/MIC correlated with treatment success at EOT (Tables 2 and 3). The MIC-adjusted correlations were stronger when compared with MIC-unadjusted equivalents (Table 3).

**Table 2.** Univariate Generalized Logistic Regression for Treatment Failure (Left Columns) and Cox-Proportional Hazard for Recurrence (Right Columns)

	Treatment Failure					Recurrence				
	*N*	OR	2.5%	97.5%	*P*-value	*N*	OR	2.5%	97.5%	*P*-value
Clinical covariates										
Adherence	95	4.25e+00	1.98e-01	3.66e+01	.287000	89	0.00000	0.00e+00	Inf	.9990
Age	95	1.00e+00	8.86e-01	1.12e+00	.955000	89	0.95600	8.38e-01	1.09e+00	.5090
Body weight	92	1.12e+00	9.85e-01	1.29e+00	.084600	86	1.00000	8.68e-01	1.15e+00	.9950
Sex	95	2.46e+07	0.00e+00	NA	.069200	89	1.30000	1.35e-01	1.25e+01	.8190
HIV	95	1.15e+00	1.82e-01	9.03e+00	.883000	89	2.37000	2.47e-01	2.28e+01	.4550
Alcohol	95	1.48e+00	1.87e-01	9.39e+00	.683000	89	2.46000	3.46e-01	1.75e+01	.3680
Smoking	95	4.79e+00	5.83e-01	3.22e+01	.131000	89	2.74000	2.85e-01	2.64e+01	.3820
Pharmacokinetic covariates										
log(Isoniazid AUC_0–24_)	95	8.88e-02	4.80e-03	1.10e+00	.060400	89	0.77400	7.26e-02	8.25e+00	.8320
log(Isoniazid C_MAX_)	95	1.02e-03	7.00e-07	4.45e-01	.025000	89	0.20900	6.25e-04	7.00e+01	.5980
log(Rifampicin AUC_0–24_)	95	8.95e-02	1.70e-03	1.86e+00	.130000	89	3.69000	4.15e-01	3.28e+01	.2420
log(Rifampicin C_MAX_)	95	7.95e-01	1.69e-02	2.38e+01	.902000	89	1.09000	2.50e-02	4.75e+01	.9640
log(Pyrazinamide AUC_0–24_)	95	1.30e-01	1.98e-03	4.45e+00	.277000	89	1.95000	6.65e-02	5.69e+01	.6990
log(Pyrazinamide C_MAX_)	95	8.10e-02	1.85e-04	2.28e+01	.393000	89	2.20000	6.25e-03	7.77e+02	.7920
log(Ethambutol AUC_0–24_)	95	2.57e-03	1.00e-06	1.22e+00	.058600	89	6.12000	2.71e-02	1.38e+03	.5120
log(Ethambutol C_MAX_)	95	3.51e-02	1.22e-04	4.01e+00	.174000	89	0.04480	1.53e-04	1.32e+01	.2840
Static bacteriological covariates										
log10(Observed baseline sputum bacillary load [CFU/ml])	91	1.68e+00	9.00e-01	3.87e+00	.109000	85	0.85700	4.82e-01	1.52e+00	.5990
MGIT negativity (month 2)	80	3.19e+00	4.97e-01	2.54e+01	.215000	74	6.54000	6.80e-01	6.29e+01	.1040
Serial Sputum Colony Counting negativity (month 2)	78	9.33e+01	1.03e+01	2.19e+03	.000036	72	0.00000	0.00e+00	Inf	.9990
Smear negativity (month 2)	89	1.25e+00	6.14e-02	9.28e+00	.850000	83	0.00000	0.00e+00	Inf	.9990
PKPD treatment response parameters										
iLAM (10e^-02^)	95	1.73e-02	2.64e-04	4.96e-01	.016200	89	0.00216	1.64e-05	2.85e-01	.0137
iT12	95	9.83e-01	9.49e-01	9.98e-01	.074800	89	1.00000	9.98e-01	1.00e+00	.9420
iBETA	95	1.13e+06	1.09e+02	6.70e+11	.001400	89	43.40000	1.40e-02	1.35e+05	.3580

iLAM, individual estimated LAM (10e^-02^); iBETA, individual estimated BETA; iT12, individual estimated T12.

**Table 3.** AUC/MIC and C_MAX_/MIC Univariate Generalized Logistic Regression for Treatment Failure (Left Columns) and Cox-Proportional Hazard for Recurrence (Right Columns)

	Treatment Failure					Recurrence				
	*N*	OR	2.5%	97.5%	*P*-value	*N*	OR	2.5%	97.5%	*P*-value
MIC adjusted pharmacokinetic covariates										
log(Isoniazid AUC_0–24_/MIC)	47	0.01740	1.25e-04	0.2840	.001410	42	4.07000	1.44e-01	115	.410
log(Isoniazid C_MAX_/MIC)	47	0.00040	0.00e+00	0.0833	.000293	42	9.19000	1.49e-02	5660	.499
log(Rifampicin AUC_0–24_/MIC)	47	0.25100	3.99e-02	1.3000	.099900	42	8.12000	4.41e-01	149	.159
log(Rifampicin C_MAX_/MIC)	47	0.37300	6.18e-02	2.0500	.248000	42	3.16000	5.57e-02	179	.577
log(Ethambutol AUC_0–24_/MIC)	47	0.34200	2.85e-02	4.0600	.377000	42	34.60000	7.60e-03	157000	.410
log(Ethambutol C_MAX_/MIC)	47	0.23900	2.01e-02	2.6000	.227000	42	1.30000	9.43e-03	179	.917
Pharmacokinetic covariates										
log(Isoniazid AUC_0–24_)	47	0.08300	2.01e-03	1.4000	.089500	42	2.34000	4.63e-02	118	.671
log(Isoniazid C_MAX_)	47	0.00132	5.00e-07	0.7050	.037800	42	0.72100	1.57e-05	33200	.952
log(Rifampicin AUC_0–24_)	47	0.35700	7.33e-03	5.0600	.499000	42	7.54000	2.68e-01	212	.235
log(Rifampicin C_MAX_)	47	1.79000	2.18e-02	62.3000	.770000	42	1.50000	1.47e-03	1530	.909
log(Ethambutol AUC_0–24_)	47	0.01630	4.20e-06	9.6900	.220000	42	12.90000	2.24e-04	742000	.648
log(Ethambutol C_MAX_)	47	0.00267	8.00e-07	1.1500	.056000	42	0.00015	0.00e+00	495	.250

From the dynamic measurements of treatment response, lower model-derived LAM and higher model-derived BETA correlated with failure at EOT (*P < .*05) on univariate generalized logistic regression (Table 2). Lower LAM was also the only variable from any analysis that associated with TB recurrence (*P < *.05) (Tables 2 and 3).

## DISCUSSION

The *C*_MAX_ and AUC_0-24_ estimates for each drug from our PK models were consistent with prior studies in African populations [14, 19–21]. Rifampicin exposure was low, supporting the ongoing case for increasing current rifampicin dosages for TB treatment [22]. Our PKPD model adequately captured the typical bacillary clearance profile and demonstrated that interindividual variability in antibiotic exposure influences treatment response. Early bacterial clearance (characterized by the LAM parameter) is faster with higher isoniazid AUC_0-24_, and bacillary clearance remains rapid, with later progression to biphasic decline, with higher rifampicin AUC_0-24_. These findings are consistent with prior reports that the bactericidal effect of isoniazid is enhanced by coadministration with rifampicin [6]. Although the model structure was rather empirical in its description of decreasing bactericidal effect over time, we did not consider more complex mechanistic PKPD models to avoid over parameterization [23].

Elevated serum bilirubin levels have previously been reported with prolongation of isoniazid clearance [24] and may also correlate with higher exposure of the other 2 hepatically cleared drugs rifampicin and pyrazinamide. This may be considered as a partial measure of the ability of the body to clear these antibiotics.

Static bacteriological measurements (eg, sputum smear or culture conversion at month 1 or 2) are commonly used to monitor TB treatment response in clinical practice, and early phase clinical trials and in this study SSCC at month 2 predicted final outcome. Some larger analyses of pooled individual patient data have described a similar relationship between month 2 culture results and outcome, although it has also been noted that correlations are not predictive at individual patient level [5, 12].

The MIC-adjusted PK estimates, ie, AUC_0-24_/MIC or *C*_MAX_/MIC, have been reported as marker of the sterilizing effect of anti-TB drugs; in line with these findings, our PKPD model displayed significant correlations in a subset of patients (Supplementary S3 Table) [25]. It showed significant correlations between isoniazid *C*_MAX_, *C*_MAX_/MIC, and AUC_0-24_/MIC and treatment success at EOT, but not with remaining free of TB (Table 3).

Caution is required when interpreting the findings that isoniazid *C*_MAX_ correlates separately with LAM and with treatment failure; colinearity between explanatory variables on univariate analyses could bias interpretation of the results where isoniazid drives early bacillary clearance from sputum, which subsequently correlates with treatment failure. Multivariate regression sometimes resolves such issues, but it was not feasible in this dataset of 95 patients with only 5 failures. Overall, delineation of a relationship between antibiotic PK and treatment response remains noteworthy. It underlines the role of PK data in explaining trial results and indicates that treatment shortening decisions for individual patients require consideration of antibiotic exposure at steady state, eg, 2 months. A large recent meta-analysis seeking to define an “easy-to-treat” patient phenotype explicitly commented on the lack of PK data for assessment [26].

Our dynamic, model-derived, parameters of bacterial clearance over the first 8 weeks of treatment correlated with treatment failure at EOT (LAM and BETA) and recurrence (LAM). These results indicate that the pattern of early bacillary clearance matters. The finding that LAM was the only predictor of relapse suggests that efficient use of quantitative longitudinal data at the level of individual patients may be preferable to reliance on traditional static measurements of bacteriological response [27].

Our study has limitations. Isolates from recurrent TB infections were not sequenced and consequently it remained unclear whether these were reinfections or relapses. Moreover, the PKPD model remains descriptive rather than predictive, due to the inability to split data into training and test batches. Antimicrobial concentration ranges were restricted by standard dosing guidelines, yet a concentration-effect relationship could be characterized. However, the model is not suitable for extrapolations and dose optimizations due to the absence of a concentration-effect relationship that was characterized over an extended concentration range. The MIC data were not available for every patient and were not available at all for pyrazinamide, hindering efforts to account for the variability added to PKPD models by incorporating MIC measurements to indices such as AUC_0-24_/MIC or *C*_MAX_/MIC [25]. More comprehensive MIC data may be of particular importance in global settings where clinical *Mtb* isolates are less uniformly antibiotic sensitive than in Malawi.

A large pooled individual patient data analysis, ideally with dose escalating studies, would enable further characterization of early bacillary clearance from sputum and provide improved statistical power to correlate early bacillary clearance with treatment failure at EOT and recurrence. Nonetheless, the data presented here illustrate the value of PKPD and dynamic bacillary clearance modeling techniques that could be applicable beyond the drug combination used in this study. A more extensive use of this approach may improve regimen evaluation in clinical trials. Moreover, it could help generate algorithms to assist with clinical decision making for individualized or stratified treatment strategies. These algorithms can take into account how well the patient is doing during early phases of treatment rather than just baseline measures on how well the patients is before treatment starts.

## CONCLUSIONS

In conclusion, isoniazid and rifampicin exposure correlate with bacillary clearance from sputum during the first 8 weeks of treatment for pulmonary TB, and bacillary clearance correlates with clinical outcome. A pooled individual patient data analysis is needed to validate the range of early PK and sputum bacillary clearance effects that predict treatment failure and relapse.

## Supplementary Material

ofaa218_suppl_Supplementary_Material
