# Supplementary material for: Longitudinal Pharmacokinetic-Pharmacodynamic Biomarkers Correlate With Treatment Outcome in Drug-Sensitive Pulmonary Tuberculosis: A Population Pharmacokinetic-Pharmacodynamic Analysis
Source: Open Forum Infect Dis. 2020 Jun 6;7(7):ofaa218. doi: 10.1093/ofid/ofaa218 (PMC7378673; doi:10.1093/ofid/ofaa218)
Supplement: ofaa218_suppl_Supplementary_Material [file ofaa218_suppl_supplementary_material.pdf]

SUPPLEMENTARY MATERIALS: Longitudinal Pharmacokinetic-Pharmacodynamic biomarkers correlate with treatment outcome in drug sensitive pulmonary tuberculosis; a population pharmacokinetic-pharmacodynamic analysis

Contents

1 S1 Fig.: Consort diagram **2**

2 Further pharmacokinetic methods **3**

3 Further pharmacokinetic results **4**

3.1 S1 Table: Summary of isoniazid, rifampicin, pyrazinamide and ethambutol population pharmacokinetic parameter estimates. 4

3.2 S2 Fig.: Basic goodness of fit plots pharmacokinetic models. 5

3.3 S3 Fig.: Stratified pharmacokinetic visual predictive checks 6

4 Further pharmacokinetic-pharmacodynamic methods **10**

5 Further pharmacokinetic-pharmacodynamic results **11**

5.1 S2 Table: Summary of population pharmacokinetic-pharmacodynamic parameter estimates. . 11

5.2 S4 Fig.: Basic goodness of fit plots pharmacokinetic-pharmacodynamic model. 12

5.3 S5 Fig.: Stratified pharmacokinetic-pharmacodynamic visual predictive checks 13

5.4 S6 Fig.: AUC-AUC/MIC and C_{MAX}-CMAX~/MIC correlation 14

5.5 S3 Table: Comparison of AUC_{INH} vs. AUC_{INH}/MIC and AUC_{RIF} vs. AUC_{RIF}/MIC 15

References **16**

1 S1 Fig.: Consort diagram

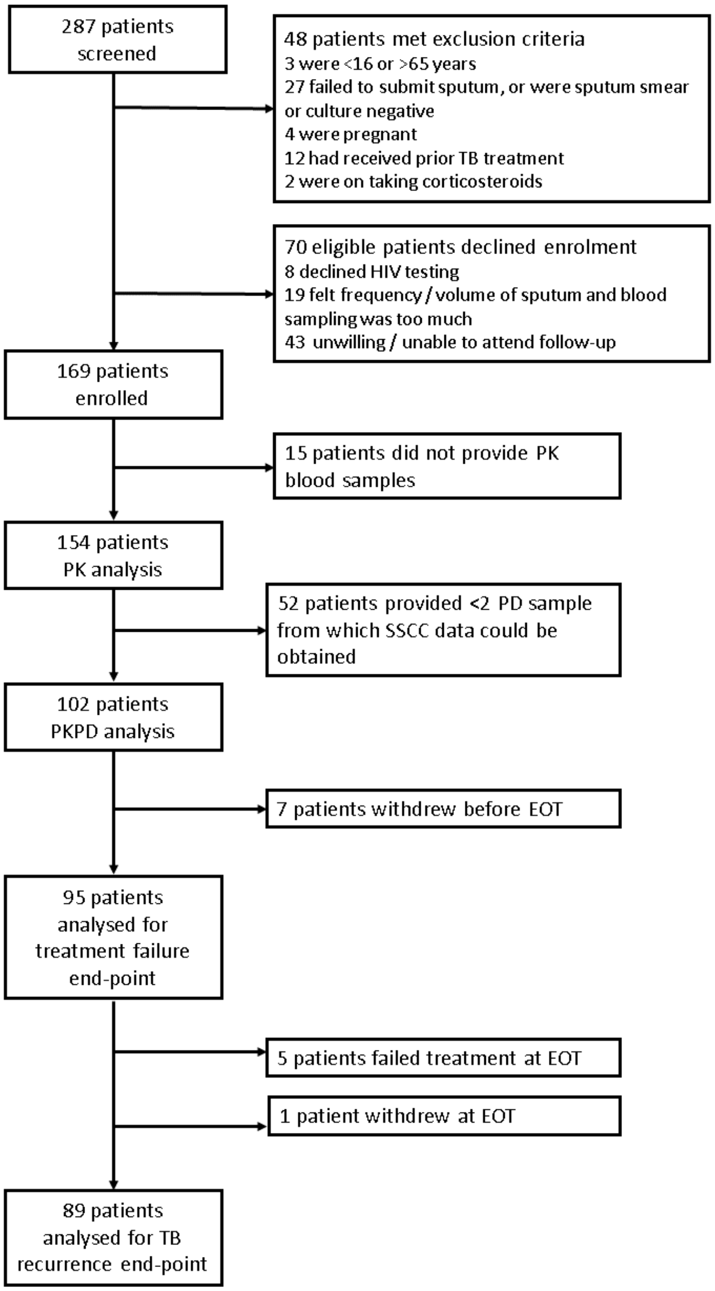

2 Further pharmacokinetic methods

Pharmacokinetic parameters were MU-transformed:

$$P_i = e^{log(\theta)+\eta}$$

Individual parameter estimates (P_i) constituted a typical population estimate (θ) and random between patient variability (η). The Iterative Two Stage estimation method was used to identify intial values for the parameter search, stochastic approximation expectation maximization was used to estimate parameters and importance sampling was used to calculate the objective function (-2 log-likelihood; -2LL) and the covariance matrix.

Discrimination between two hierarchical models was based on changes in the objective function value (-2LL, with drops of more than 3.84 or 6.63 points considered significant at $p<0.05$ or $p<0.01$ for the inclusion of 1 degree of freedom), precision in parameter estimates (relative standard error, RSE %, and confidence intervals, calculated as 2.5 and 97.5 percentile of the data, both derived from 1,000 non-parametric bootstraps), graphical analysis of model accuracy and prediction (e.g. goodness of fit plots and visual predictive checks), and physiological and micro-biological plausibility. Goodness of fit plots comprise graphical representations of observed vs. individual level model predictions and normalized predictive distribution errors vs. population level model predictions. Visual predictive checks comprise 2,000 newly simulated studies using the developed model. 90% confidence intervals around the simulated 2.5, 50th and 97.5 percentiles are overlayed with the observed 2.5, 50th and 97.5 and percentiles and observations.

Parameter estimates were based on two compartment disposition models for isoniazid and ethambutol and one compartment disposition models for rifampicin and pyrazinamide. Rifampicin and ethambutol absorption were described using transit absorption models. Isoniazid and pyrazinamide absorption were described using first-order absorption models. Bodyweight was incorporated as a covariate on clearance and volume estimates using allometry, centralised around 70 kg patient for rifampicin and pyrazinamide and 63 kg and 50 kg for isoniazid and ethambutol. Rifampicin clearance estimates were centralised around male patients. Clearance and apparent distribution volume of the central compartment were estimated with absorption, peripheral distribution volume and inter-compartment clearance fixed to isoniazid [1], rifampicin [2], pyrazinamide [3] and ethambutol [4] literature values. This approach supported characterisation of the entire pharmacokinetic profiles (i.e. absorption, distribution and elimination) which was not possible based on the study data alone. For isoniazid, Q, V_P and k_a in the population pharmacokinetic model were fixed to literature values from a healthy volunteer population [1] as fixing parameter estimates to literature values from a patient population [5] resulted in unrealistically long terminal half-lives. Moreover, isoniazid elimination clearance parameters were not accounted for NAT2 acetylator status. Residual variability was described using proportional error model for isonizaid, rifampicin and ethambutol and a combined proportional/additive model for pyrazinamide.

3 Further pharmacokinetic results

3.1 S1 Table: Summary of isoniazid, rifampicin, pyrazinamide and ethambutol population pharmacokinetic parameter estimates.

	isoniazid	rifampicin	pyrazinamide	ethambutol
Cl (l/hr)	13.70	16.90	3.86	45.50
Cl CI	(12.8-14.88)	(14.73-20.16)	(3.66-4.04)	(43.47-47.6)
Vc (l)	39.7	31.3	45.2	124.0
Vc CI	(34.68-45.43)	(23.19-39.06)	(43.47-47.5)	(109.26-142.45)
Q (l/hr)	2.9 fixed	-	-	34.3 fixed
Vp (l)	16.5 fixed	-	-	623 fixed
ka (hr-1)	0.6 fixed	0.277 fixed	3.94 fixed	0.474 fixed
MTT (hr)	-	0.326 fix	-	0.789 fix
Transit compartments (n)	-	1.5 fix	-	-
Cl~Male	-	0.183	-	-
Cl~Male CI	-	(-0.036 - 0.385)	-	-
IIV on Cl (%CV)	46.66	40.08	28.60	20.99
IIV on Cl CI (%CV)	(38.92-54.58)	(31.21-49.73)	(23.41-33.02)	(15.62-25.57)
IIV on Vc (%CV)	-	84.52	3.95	-
IIV on Vc CI (%CV)	-	(54.22-133.21)	(0.22-11.48)	-
IIV on ka (%CV)	36.64 fixed	-	397.2	67.56 fixed
IIV on ka CI (%CV)	-	-	(174.38-1005.27)	-
IIV on MTT (%CV)	-	27.05 fix	-	109.6 fix
Proportional residual variability (%)	21.10	19.40	3.06	12.80
Proportional residual variability CI (%)	(17.87-24.56)	(15.18-23.67)	(0.38-5.21)	(9.85-15.92)
Additive residual variability	-	-	74.5	-
Additive residual variability CI	-	-	(12.65-462.32)	-
Cmax (mg/l)	3.24 [2.19-5.5]	4.35 [2.3-12.3]	40 [25-63]	2.3 [1.43-4.4]
AUC (hrxmg/l)	18.83 [6.97-71.2]	29.1 [15.4-119]	419 [210-1014]	18.5 [12.8-38.5]

CL: elimination clearance, V_C: apparant volume of central compartment, Q: inter-compartmental clearance, V_P: apparent colume of peripheral compartment, k_a: absorption rate constant, MTT: mean transit time, IIV: inter individual variability, C_{max}: maximum concentration, AUC: area under the concentration-time curve and CI: Confidence Interval. IIV was calculated as $100 \times \sqrt{e^{\eta} - 1}$.

3.2 S2 Fig.: Basic goodness of fit plots pharmacokinetic models.

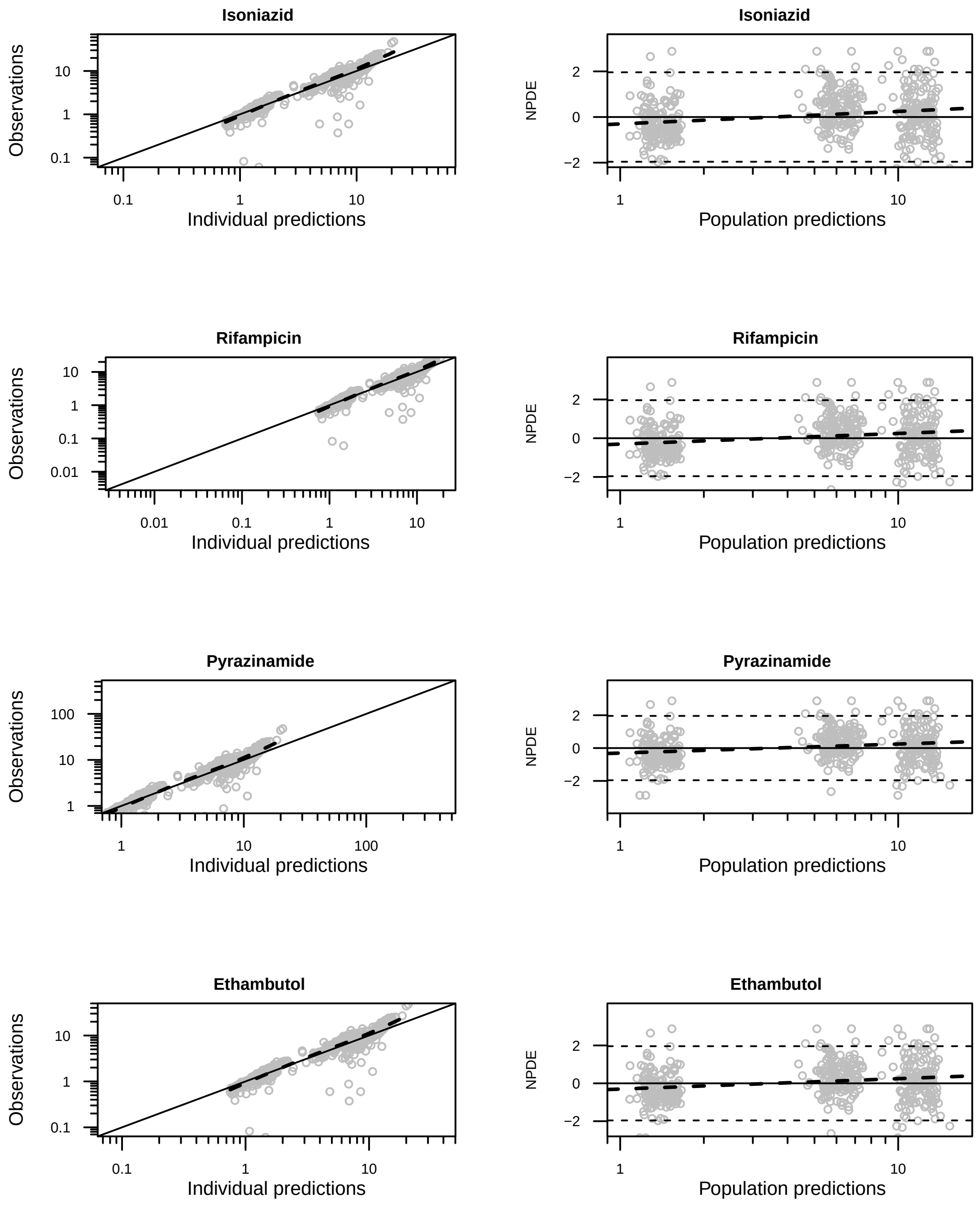

Observed-individual predictions and Normalised-Prediction Distribution Errors (NPDE)-population predictions plots for isoniazid, rifampicin, pyrazinamide and ethambutol. Grey open circles represent the observations, the solid and dashed black lines represent the unity lines and the 95% of the Gaussian distribution. The bold dashed black line represent a local polynomal regression fitting.

3.3 S3 Fig.: Stratified pharmacokinetic visual predictive checks

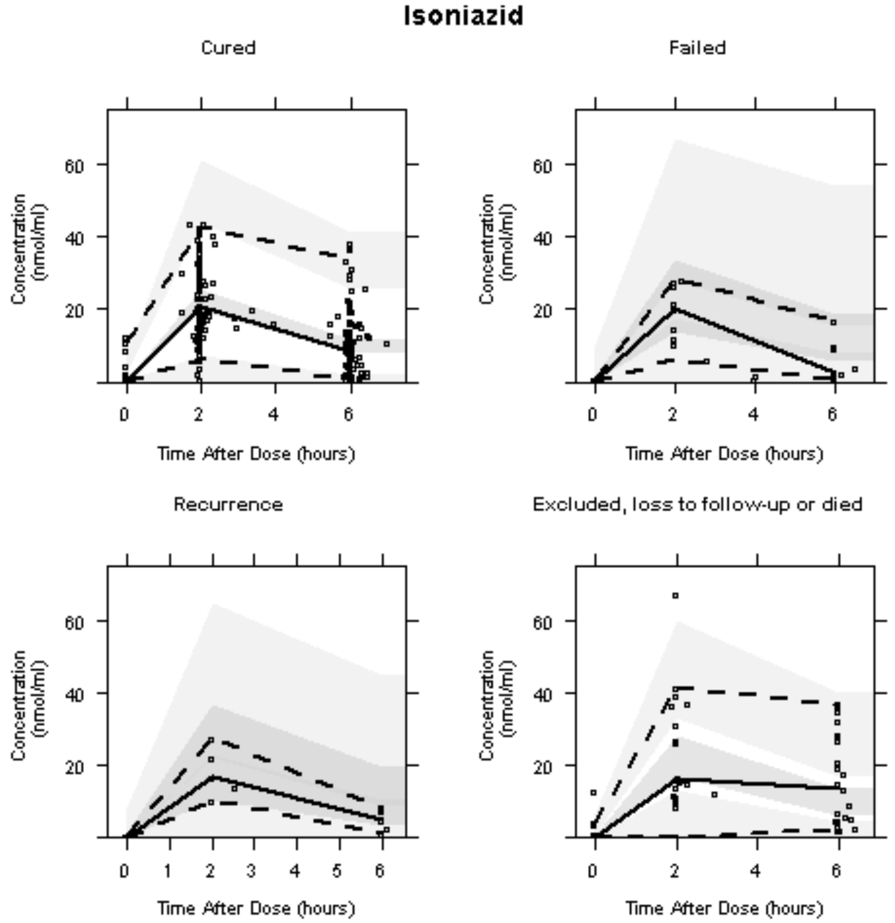

Simulation based (n=2,000) VPCs for isoniazid, stratified by treatment outcome. Open circles represent observations, solid and dashed black lines represent observed 2.5, 50th and 97.5 percentiles. Shaded areas represent the 90% confidence intervals around the simulated 2.5, 50th and 97.5 percentiles. Cured: patients that had no recurrent TB during 12 month follow-up after cure at EOT, Failed: patients that failed treatment at EOT, Recurrence: patients that had a recurrent infection during the 12 month follow-up after cure at EOT, and excluded, loss to follow-up or died: patients that were excluded for treatment failure analysis, were loss to or did not enther the follow-up and patients that died.

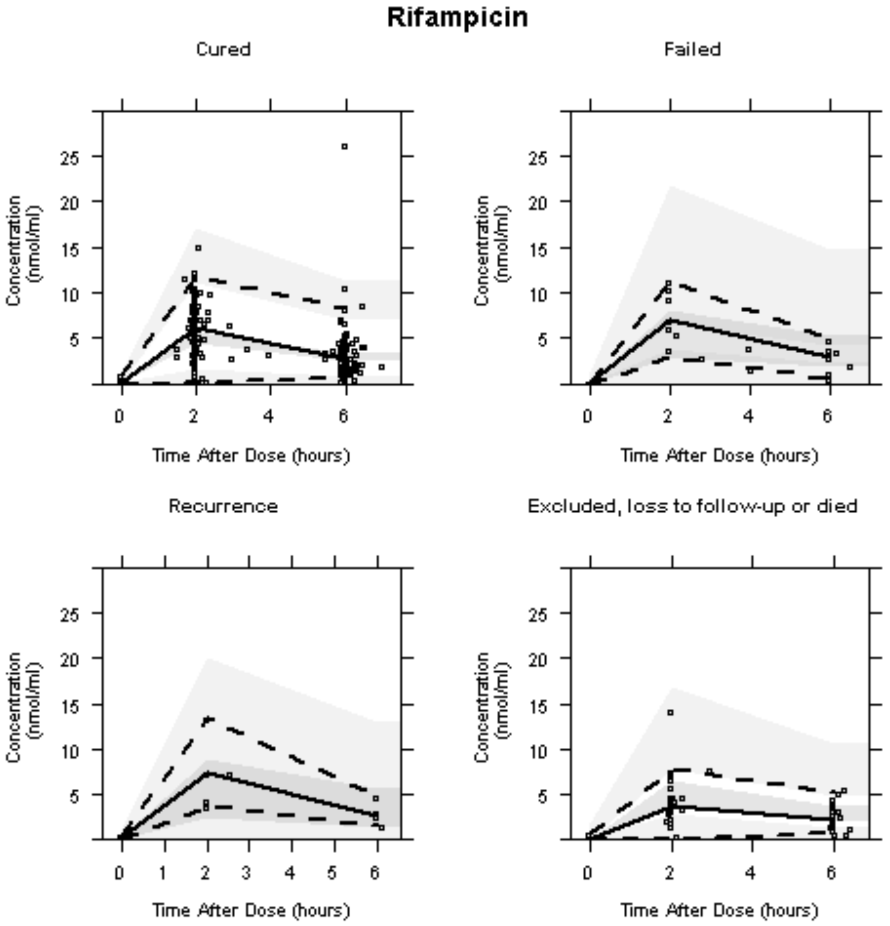

Simulation based (n=2,000) VPCs for rifampicin, stratified by treatment outcome. Open circles represent observations, solid and dashed black lines represent observed 2.5, 50th and 97.5 percentiles. Shaded areas represent the 90% confidence intervals around the simulated 2.5, 50th and 97.5 percentiles. Cured: patients that had no recurrent TB during 12 month follow-up after cure at EOT, Failed: patients that failed treatment at EOT, Recurrence: patients that had a recurrent infection during the 12 month follow-up after cure at EOT, and excluded, loss to follow-up or died: patients that were excluded for treatment failure analysis, were loss to or did not enther the follow-up and patients that died.

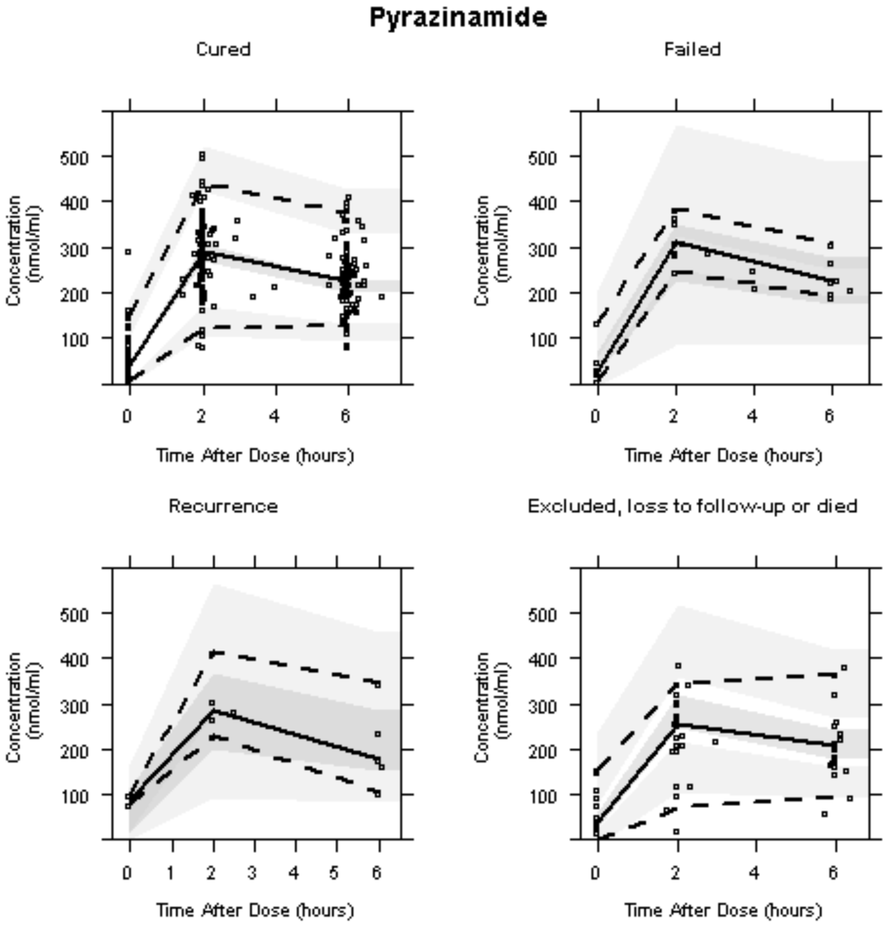

Simulation based (n=2,000) VPCs for pyrazinamide, stratified by treatment outcome. Open circles represent observations, solid and dashed black lines represent observed 2.5, 50th and 97.5 percentiles. Shaded areas represent the 90% confidence intervals around the simulated 2.5, 50th and 97.5 percentiles. Cured: patients that had no recurrent TB during 12 month follow-up after cure at EOT, Failed: patients that failed treatment at EOT, Recurrence: patients that had a recurrent infection during the 12 month follow-up after cure at EOT, and excluded, loss to follow-up or died: patients that were excluded for treatment failure analysis, were loss to or did not enther the follow-up and patients that died.

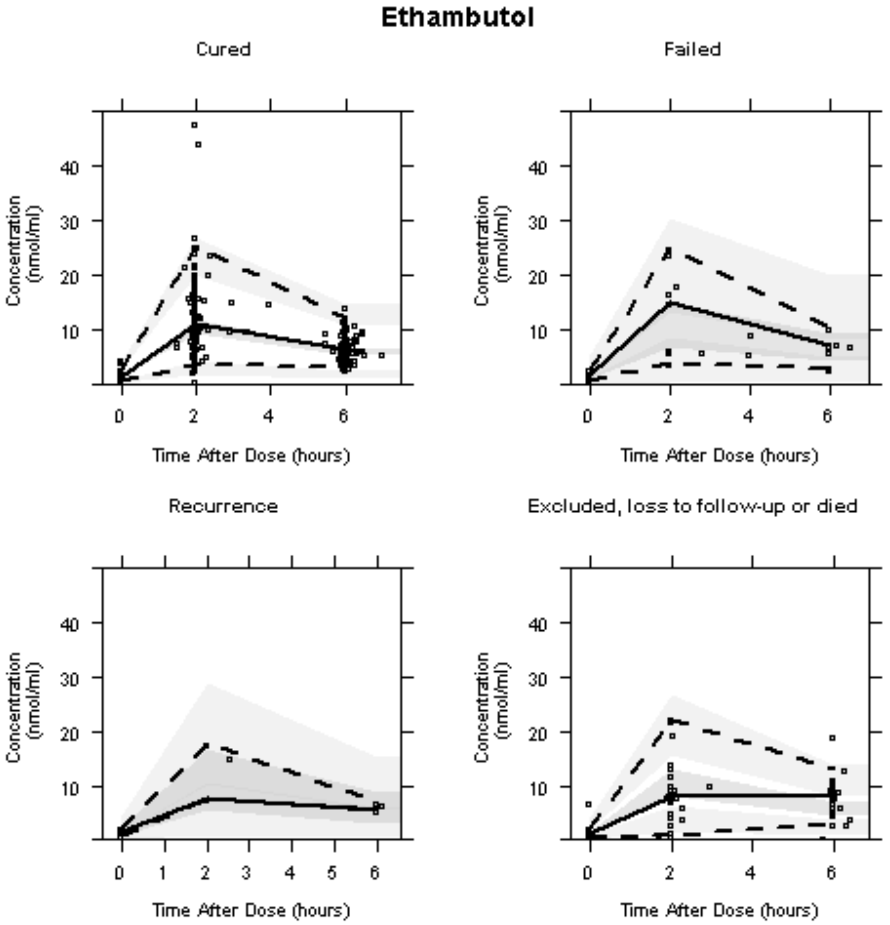

Simulation based (n=2,000) VPCs for ethambutol, stratified by treatment outcome. Open circles represent observations, solid and dashed black lines represent observed 2.5, 50th and 97.5 percentiles. Shaded areas represent the 90% confidence intervals around the simulated 2.5, 50th and 97.5 percentiles. Cured: patients that had no recurrent TB during 12 month follow-up after cure at EOT, Failed: patients that failed treatment at EOT, Recurrence: patients that had a recurrent infection during the 12 month follow-up after cure at EOT, and excluded, loss to follow-up or died: patients that were excluded for treatment failure analysis, were loss to or did not enther the follow-up and patients that died.

4 Further pharmacokinetic-pharmacodynamic methods

Population PKPD models were developed using the ADVAN6 subroutine NONMEM and colony count number below the limit of quantification were handled with the M3 method. [6] Sputum bacillary load data above the limit of quantification was estimated and the likelihood that sputum bacillary load data was below the limit of quantification was maximised which is the standard procedure to account for data below limit of quantification. Except for $\mathrm{CFU_{baseline}}$, which followed normal distribution as the power of base 10 was estimated, all other PKPD parameters followed log-normal distribution:

$$P_i = \theta e^{\eta}$$

Individual parameter estimates (P_i) constituted a typical population estimate (θ) and random between patient variability (η). A single differential equation and a proportional bacterial killing rate ($\mathrm{k_{net}}$) was used to describe the sputum bacillary load data with baseline sputum bacillary load as initial condition estimated:

$$\frac{dB}{dt} = -k_{net}B$$

Effective bacterial killing on $\log_{10}$ transformed displayed a bi-phasic distribution in some cases which was accomodated by an exponential model:

$$k_{net} = -\theta_{LAM}(1 - \theta_{\beta} * (1 - e^{-time\frac{LN(2)}{\theta_{T_{1/2}}}}))$$

The time dependency in the equation was represented by $\theta_{T_{1/2}}$, the magnitude of decreasing early bacillary clearance over time by θ_{β} and the underlying sputum bacillary effect by θ_{LAM}. Criteria to discriminate between two hierarchical PKPD models were identical to the criteria used for the development of pharmacokinetic models.

All covariate-parameter relations for continuous variables were evaluated using *linear* $(1+(\theta(COV-median)))$, *exponential* $(e^{\theta(COV-median)})$ and *power* $(\frac{COV}{median}^{\theta})$ equations and AUC and $\mathrm{C_{MAX}}$ parameters were also evaluated using an E_{MAX} model $(\frac{COV}{COV+EC_{50}})$. Categorical variables were evaluated using a *proportional* $((1+\theta)$ equation.

The same criteria as described under the further pharmacokinetic method sections were used for discrimination between two hierarchical models.

5 Further pharmacokinetic-pharmacodynamic results

5.1 S2 Table: Summary of population pharmacokinetic-pharmacodynamic parameter estimates.

	Estimate	BSestimate	CI
BL (log10 CFU/ml)	6.3867	6.3867	(6.3864 - 6.3876)
LAM (CFU/ml per hour)	0.0389	0.0389	(0.0389 - 0.0389)
T1/2 (hours)	149.5800	149.5668	(149.571 - 149.594)
BETA [0,1]	0.6445	0.6444	(0.6444 - 0.6446)
LAM_alcohol	-0.0377	-0.0377	(-0.0378 - -0.0377)
LAM_AUCinh	0.0005	0.0001	(-0.0014 - 5e-04)
LAM_BLbilirubin	0.0029	0.0031	(-0.0063 - 0.0135)
T12_AUCrif	0.0335	0.0331	(0.0179 - 0.049)
BL_var	0.1135	0.1135	(0.1135 - 0.1136)
OMEGA.2.1.	0.0028	0.0028	(0.0028 - 0.0028)
LAM_var	0.0383	0.0383	(0.0383 - 0.0383)
OMEGA.3.1.	0.0000	0.0000	(0 - 0)
OMEGA.3.2.	-0.0037	-0.0037	(-0.0037 - -0.0037)
T1/2_var	0.0526	0.0526	(0.0526 - 0.0526)
OMEGA.4.1.	0.3846	0.3843	(0.3841 - 0.385)
OMEGA.4.2.	0.0102	0.0102	(0.0101 - 0.0102)
OMEGA.4.3.	-0.0085	-0.0085	(-0.0085 - -0.0085)
BETA_var	1.3149	1.3132	(1.3115 - 1.3163)
RUV	2.5832	2.5832	(2.5826 - 2.5834)

BL: estimated baseline bacillary load, LAM: bacterial clearance rate, $T_{1/2}$: start time of delayed bacillary clearance, BETA: magnitude of decreased bacillary clearance, LAM_alcohol: effect of alcohol consumption on LAM, LAM_AUCinh: effect of $AUC_{isoniazid}$ of LAM, LAM_BLbilirubin: effect of baseline bilirubin on LAM, T12_AUCrif: effect of $AUC_{rifampicin}$ on start time of delayed bacillary clearance. Between patient variability was estimated in a block with OMEGA's being the off-diagnals and parameter names with "_var" being the diagnoal estimates. RUV: additive residual variability on $\log_{10}$ transformed data.

5.2 S4 Fig.: Basic goodness of fit plots pharmacokinetic-pharmacodynamic model.

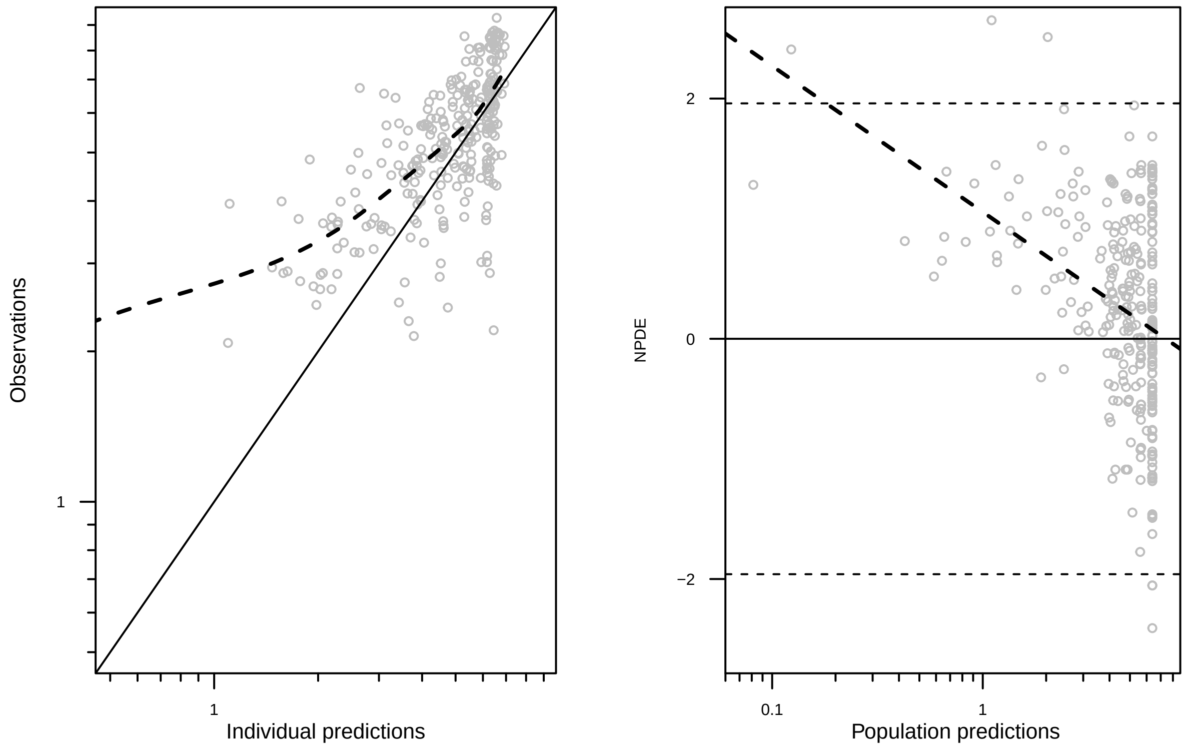

Observed-individual predictions and Normalised-Prediction Distribution Errors (NPDE)-population predictions plots for the PKPD model. Grey open circles represent the observations, the solid and dashed black lines represent the unity lines 95% of the Gaussian distribution. The bold dashed black line represent a local polynomal regression fitting.

5.3 S5 Fig.: Stratified pharmacokinetic-pharmacodynamic visual predictive checks

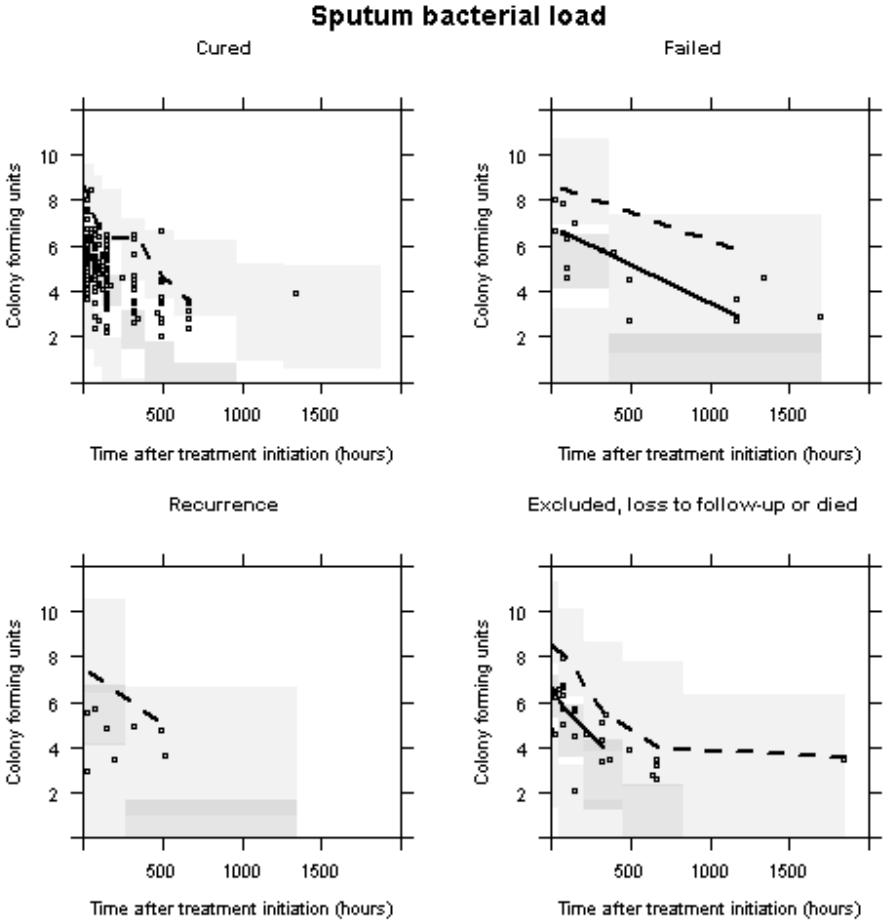

Simulation based (n=2,000) VPCs for sputum bacterial load, stratified by treatment outcome. Open circles represent observations, solid and dashed black lines represent observed 2.5, 50th and 97.5 percentiles. Shaded areas represent the 90% confidence intervals around the simulated 2.5, 50th and 97.5 percentiles. Cured: patients that had no recurrent TB during 12 month follow-up after cure at EOT, Failed: patients that failed treatment at EOT, Recurrence: patients that had a recurrent infection during the 12 month follow-up after cure at EOT, and excluded, loss to follow-up or died: patients that were excluded for treatment failure analysis, were loss to or did not enther the follow-up and patients that died.

5.4 S6 Fig.: AUC-AUC/MIC and C_{MAX}-CMAX~/MIC correlation

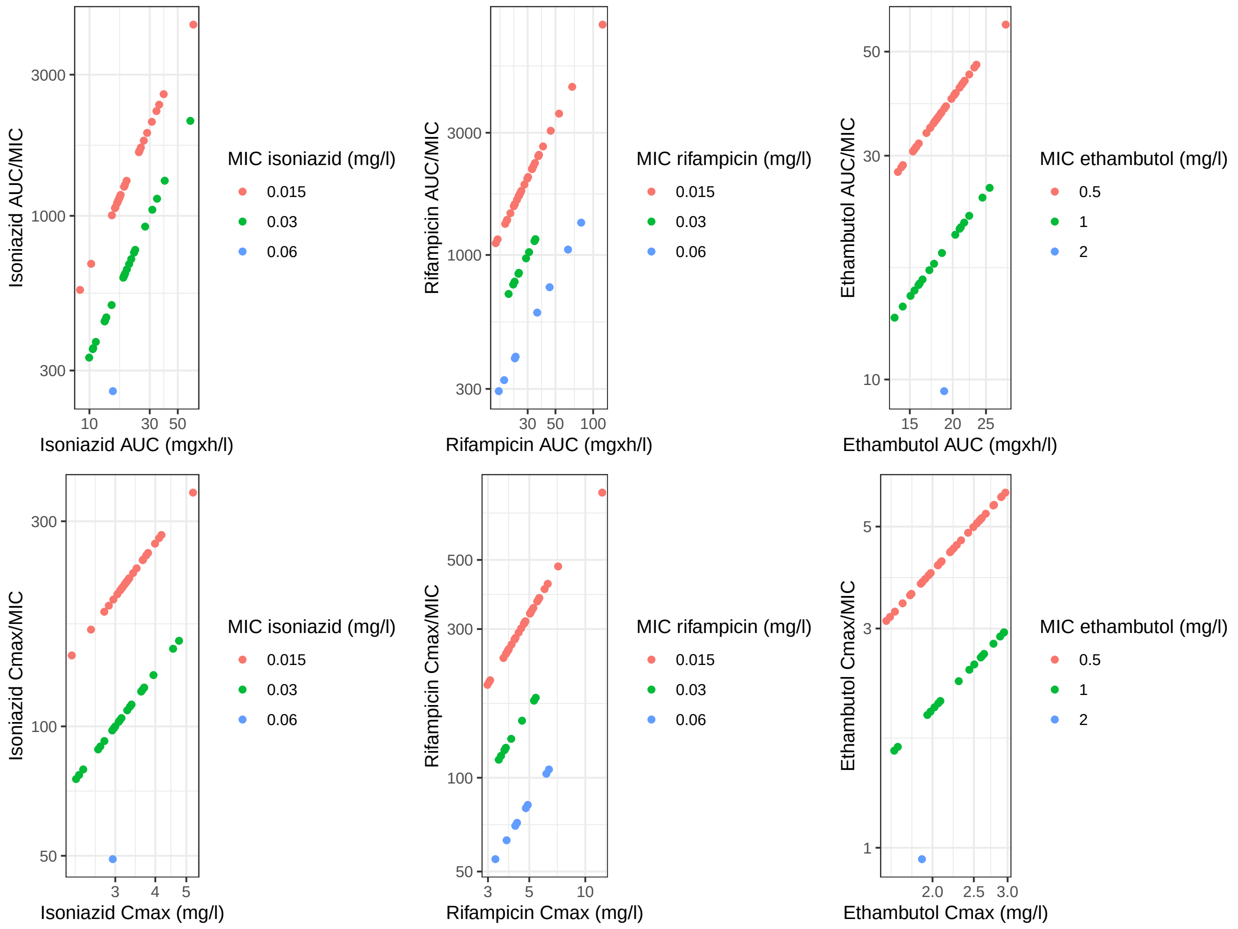

5.5 S3 Table: Comparison of AUC_{INH} vs. AUC_{INH}/MIC and AUC_{RIF} vs. AUC_{RIF}/MIC

Model	OFV	ΔOFV	p-value
Final AUC/MIC instead of AUC	433.193	-	-
-	-	-	-
AUC_{INH}/MIC omitted	455.459	22.266	<0.01
-	-	-	-
AUC_{RIF}/MIC omitted	-	-	-
-	-	-	-

References

1. Seng K-Y, Hee K-H, Soon G-H, Chew N, Khoo SH, Lee LS-U. Population pharmacokinetic analysis of isoniazid, acetylisoniazid, and isonicotinic acid in healthy volunteers. Antimicrobial agents and chemotherapy **2015**; 59:6791–6799.

2. Sloan DJ, McCallum AD, Schipani A, et al. Genetic determinants of the pharmacokinetic variability of rifampin in malawian adults with pulmonary tuberculosis. Antimicrobial agents and chemotherapy **2017**; 61:e00210–17.

3. Alsultan A, Savic R, Dooley KE, et al. Population pharmacokinetics of pyrazinamide in patients with tuberculosis. Antimicrobial agents and chemotherapy **2017**; 61:e02625–16.

4. Jönsson S, Davidse A, Wilkins J, et al. Population pharmacokinetics of ethambutol in south african tuberculosis patients. Antimicrobial agents and chemotherapy **2011**; 55:4230–4237.

5. Wilkins JJ, Langdon G, McIlleron H, Pillai G, Smith PJ, Simonsson US. Variability in the population pharmacokinetics of isoniazid in south african tuberculosis patients. British journal of clinical pharmacology **2011**; 72:51–62.

6. Beal SL. Ways to fit a pk model with some data below the quantification limit. Journal of pharmacokinetics and pharmacodynamics **2001**; 28:481–504.
